# Global drought trends and future projections

**DOI:** 10.1098/rsta.2021.0285

**Published:** 2022-12-12

**Authors:** Sergio M. Vicente-Serrano, Dhais Peña-Angulo, Santiago Beguería, Fernando Domínguez-Castro, Miquel Tomás-Burguera, Iván Noguera, Luis Gimeno-Sotelo, Ahmed El Kenawy

**Affiliations:** ^1^ Instituto Pirenaico de Ecología, Consejo Superior de Investigaciones Científicas (IPE–CSIC), Zaragoza 50059, Spain; ^2^ HydroSciences Montpellier, University Montpellier, CNRS, IRD, CEDEX, Montpellier 34090, France; ^3^ Estación Experimental de Aula Dei, Consejo Superior de Investigaciones Científicas (EEAD–CSIC), Zaragoza 50059, Spain; ^4^ Aragonese Agency for Research and Development Researcher (ARAID), University of Zaragoza, Zaragoza, Spain; ^5^ Department of Geography, University of Zaragoza, Zaragoza, Spain; ^6^ Centre National de Recherches Météorologiques, Université de Toulouse, Météo-France, CNRS, Toulouse 31057, France; ^7^ Centro de Investigación Mariña, Universidade de Vigo, Environmental Physics Laboratory (EPhysLab), Ourense, Spain; ^8^ Department of Geography, Mansoura University, Mansoura, Egypt

**Keywords:** precipitation, atmospheric evaporative demand, future scenarios

## Abstract

Drought is one of the most difficult natural hazards to quantify and is divided into categories (meteorological, agricultural, ecological and hydrological), which makes assessing recent changes and future scenarios extremely difficult. This opinion piece includes a review of the recent scientific literature on the topic and analyses trends in meteorological droughts by using long-term precipitation records and different drought metrics to evaluate the role of global warming processes in trends of agricultural, hydrological and ecological drought severity over the last four decades, during which a sharp increase in atmospheric evaporative demand (AED) has been recorded. Meteorological droughts do not show any substantial changes at the global scale in at least the last 120 years, but an increase in the severity of agricultural and ecological droughts seems to emerge as a consequence of the increase in the severity of AED. Lastly, this study evaluates drought projections from earth system models and focuses on the most important aspects that need to be considered when evaluating drought processes in a changing climate, such as the use of different metrics and the uncertainty of modelling approaches.

This article is part of the Royal Society Science+ meeting issue ‘Drought risk in the Anthropocene’.

## 1. Introduction

Unlike climate aridity and water scarcity, which are based on long-term water availability, the concept of drought is based on short-term conditions (from weeks to years). Droughts occur when water resources (such as those found in soils, rivers, reservoirs and aquifers) are insufficient to meet the needs of people or the environment and, as a result, have a negative impact on both [1]. As a result, drought must be measured as a relative deviation from long-term normal conditions [2], so that it can be compared across space and time between areas with different long-term climates (e.g. arid versus humid regions).

It is difficult to quantify the severity of a drought because it is determined by the effects or impacts it has on a variety of systems (agriculture, water resources, ecology, forestry, economy, etc.). Nevertheless, due to limited data availability and temporal inconsistencies, it is difficult to accurately assess drought dynamics based on impact data. Moreover, drought vulnerability can change substantially in both human and environmental systems [3–5], making it difficult to correlate changes in drought impacts with potential changes in drought severity induced by climate change. For these reasons, the objective evaluation of drought severity and its temporal variability and trend typically relies on physical metrics that isolate drought severity from the impact assessment. Nevertheless, this is also a challenging task. In this context, there is no single physical variable that can quantify drought. This is simply because although some variables (i.e. precipitation) are fundamental for drought severity assessment, there are other relevant variables (e.g. atmospheric evaporative demand [AED], evapotranspiration [ET] [6], soil moisture [7], streamflow [8] and vegetation conditions [9]). There are numerous feedbacks between these variables that influence drought evolution in various ways [10], particularly in relation to the dominant drought types [11] (meteorological, agricultural, ecological and hydrological) as well as the various socioeconomic sectors and natural systems affected by drought.

In addition, when modelling drought is attempted, some key variables are plagued by uncertainties (e.g. soil moisture, streamflow and groundwater) or data availability [12,13]. A wide range of meteorological variables are employed to monitor droughts, analyse trends and model outcomes for future scenarios [14]. Some of these variables, such as precipitation, ET and AED, may be affected by feedbacks resulting from land transformations and conditions (such as soil moisture) [15] or due to issues related to data temporal homogeneity. While droughts are caused mainly by low precipitation, they can be exacerbated by anomalies in AED [16] or ET [17]. As such, to accurately project future droughts, the use of different metrics derived from meteorological data can be a valuable tool.

The objective of this study is to assess recent trends and future projections of droughts on a global scale. For this purpose, we combined critical assessment of the recent scientific literature on the topic with original analysis based on observations and projections from various models from the recent CMIP6 experiment using different drought metrics. Both the literature reviews and the results of the data analysis are assessed critically, and some topics currently under debate by the scientific community (e.g. the role of the AED on drought severity, the use of different drought metrics and the uncertainty of projections) are discussed in depth. Current uncertainties and difficulties in making absolute statements about drought changes in recent decades and in future scenarios are discussed in the context of drought's complexity and multidimensionality.

## 2. Global drought trends

### (a) Recent evolution of meteorological droughts

Among the different meteorological variables, precipitation is indisputably what mostly determines drought occurrence and severity. Thus, the term ‘meteorological drought’ is widely used as a synonym for the precipitation deficit. For this reason, we started by determining whether droughts, quantified by precipitation data alone, exhibit long-term changes. We did this by using the standardized precipitation index (SPI) [18], which is a standardized metric enabling global comparison of drought changes. It has been used in several drought assessments because the World Meteorological Organization recommends it as the standard drought metric [19,20].

First, we carried out a long-term assessment using available precipitation data from global datasets. This then underwent quality control, reconstruction [21] and homogenization of precipitation datasets from the Global Historical Climatology Network and other European datasets [22]. From the original database of 25 590 stations, we selected those series that started in 1900 or before and contained at least 80% of valid records during 1900–2020. Data gaps were filled in with neighbouring series (a minimum of three) selected according to correlation (Pearson's *r* > 0.7) and distance (*d* < 400 km) criteria. Homogeneity testing showed 560 inhomogeneous series that were corrected using ratios between reference series and candidate stations. This approach was run automatically using CLIMATOL (https://www.climatol.eu). The final dataset contained 2732 complete and homogeneous series from 1900 to 2020. Trends from SPI series, and from series of the duration and magnitude of drought events, were calculated by means of a modified Mann-Kendall test that accounted for autocorrelation in the data [23]. The drought events were identified using various thresholds (see below).

**Figure 1.**
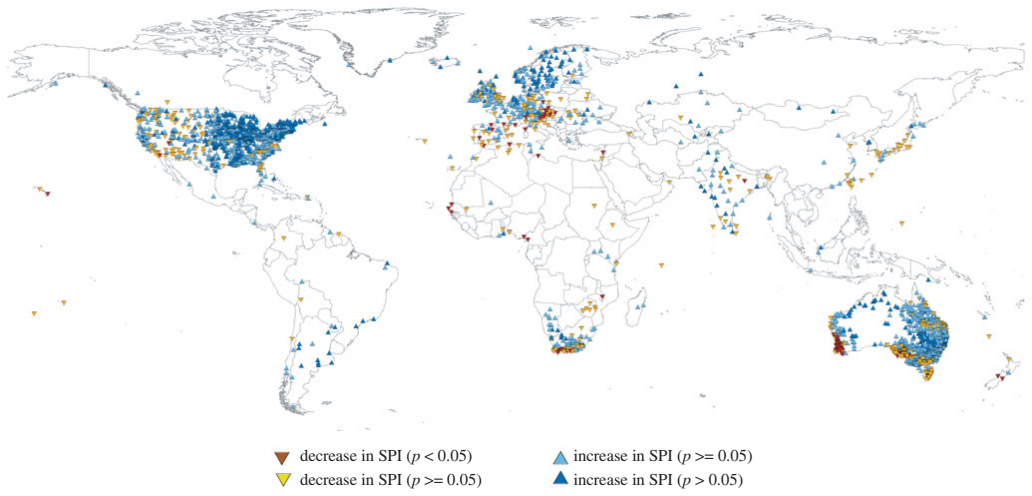
Sign and significance of the SPI annual trends (12 months in December) from 1900 to 2020 using available meteorological records worldwide. (Online version in colour.)

Figure 1 shows a predominance of positive and significant SPI trends in the 12-month SPI (January–December) for the period 1900–2020. Only a few regions showed a dominant decreasing significant trend (southwestern Australia, southern South Africa and Central Europe) informative of drying conditions. In western Africa, some meteorological stations showed negative trends, but given the small number of stations available, it is not possible to draw robust regional conclusions. In the Mediterranean region, a majority of stations did not return significant trends. Seasonally, there are some spatial differences, but again, there is no generalized decrease in SPI with a few exceptions (e.g. southern Africa in MAM, Southwestern Australia, South-eastern US and the British and Irish islands in JJA) (electronic supplementary material, figure S1). Precipitation trends were, in many cases, the result of strong decadal variability coinciding over the period of analysis. For example, the negative trends recorded in some stations across the Mediterranean by certain authors [24,25] were strongly dependent on the selected period of analysis [26], but precipitation was stationary in the long term [27].

In regard of assessing meteorological drought trends, it is interesting to consider how precipitation deficits accumulate over time in order to generate a meteorological drought event. We identified meteorological drought events in the period 1900–2020 as sequences of consecutive months with SPI values below two thresholds: −0.84, which corresponds to a return period of 1 in 5 years, and −1.65, of 1 in 20 years. This enables identification of long-term changes in mild and severe meteorological droughts, respectively. The analysis was performed using two different SPI timescales, 3 and 12 months. Patterns of change were similar to those observed in the evolution of the SPI values, since a majority of stations did not show an increase in the duration of mild and severe meteorological drought events (electronic supplementary material, figure S2). Patterns of trends in the magnitude of drought events, measured as the integral of the SPI values during the drought period, were similar (results not shown). To summarize, the analysis of both the SPI time series and meteorological drought event duration and magnitude shows a dominance of non-significant trends between 1900 and 2020 (electronic supplementary material, figure S3).

**Figure 2.**
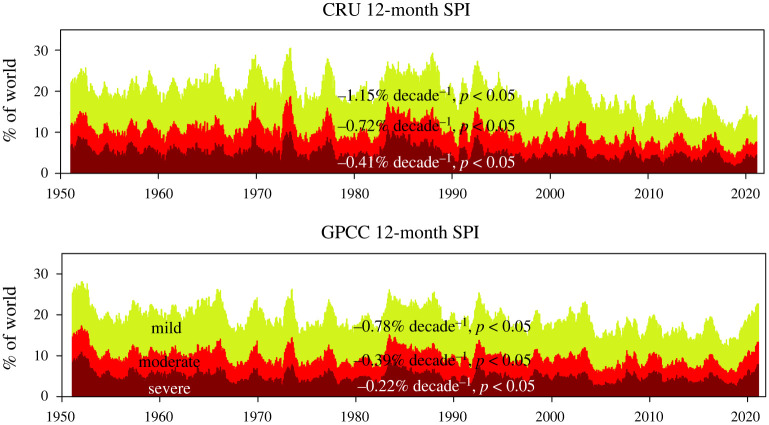
Evolution of the percentage of world surface area affected by different typologies of drought severity based on the 12-month SPI. The magnitude of change and significance of the trend were assessed with the 12-month SPI data of December (one data per year) in order to remove the effect of temporal autocorrelation that characterizes long SPI timescales. The magnitude of change was calculated by a linear regression between time (independent variable) and the percentage of global land areas affected by drought (dependent variable). (Online version in colour.)

The previous results could be biased due to the spatial distribution of the available meteorological stations, as in large regions of the world there are few or no long-term series. Therefore, we conducted further analysis using data from the middle of the twentieth century when more data became available, including gridded datasets based on the interpolation of meteorological station data. Although gridded datasets present problems for assessing long-term trends of standardized climatic variables [28], they at least provide a complete global perspective. We used monthly precipitation gridded data from the Climatic Research Unit (CRU) TS4 and the Global Precipitation Climatology Centre (GPCC) [29,30], covering the period 1950–2020. The calculation of meteorological drought events was based on a 3- and 12-month SPI and a threshold equal to zero in order to have a sufficient number of events to assess trends, as the period of analysis was shorter than in the previous case. In the vast majority of the world, trends in meteorological drought duration and magnitude are not statistically significant, with the exception of some small regions of Africa and South America, which is also where data uncertainty is greater [28] (electronic supplementary material, figure S4). Thus, if we classify the regions affected by mild (SPI < −0.84, 1 in 5 years), moderate (SPI < −1.28, 1 in 10 years) and severe (SPI < −1.65, 1 in 20 years) meteorological droughts from 1950 to 2020 and analyse the evolution of the percentage of land affected by meteorological drought, there is a statistically significant decline of the percentage of land area affected by drought conditions, which is stronger with the CRU dataset but is also observed with the GPCC dataset (figure 2).

In summary, trends in meteorological drought severity in the last few decades are not observed globally based on precipitation data, and very few areas are showing changes in the severity of meteorological droughts. Our findings agree with the assessment included in the recent AR6 IPCC report, which stresses: ‘*Trends in precipitation are not a main driver in affecting global-scale trends in drought (medium confidence), but have induced increases in meteorological droughts in a few AR6 regions*' (Chapter 11, Executive Summary, [26]). Therefore, the substantial precipitation deficits affecting different regions in the world recently [26] should be considered as merely the result of strong spatial and temporal variability in precipitation associated with the internal climate variability that drives the main atmospheric mechanisms, such as El Niño-Southern Oscillation and the North Atlantic Oscillation, which exert powerful control over meteorological drought events [31–33]. As such, reconstructions of precipitation over the past few hundred years based on documents and tree-ring data demonstrate that precipitation deficits during the pre-instrumental period were linked to potent mega-droughts [34].

Finally, we would like to stress that although temporal anomalies and changes in total precipitation amount and seasonality, quantified by means of the SPI, are the main factors controlling the occurrence of meteorological drought events and trends, there are other precipitation characteristics that are not assessed here but could also affect changes in meteorological droughts. Rainfall intensity, dry spell length and changes in effective rainfall due to land cover changes and changes in rainfall interception [35] all contribute to the assessment of recent droughts and their future projections [36,37] (see §6).

### (b) The role of atmospheric evaporative demand in the assessment of drought trends

According to the results provided in §2, if there is a notion of certain increasing trends in the severity of some drought types (i.e. agricultural, ecological and hydrological) in the last few decades, it must be related to factors other than precipitation, since meteorological droughts do not show relevant changes on a global scale. These factors can be of two types: (i) those resulting in changes in human water demands and land cover, which may increase the effects of precipitation deficits on ecosystems and human societies and reinforce the negative consequences of agricultural, ecological and hydrological droughts [38] and (ii) those resulting from anthropogenic forcing as a consequence of the enhanced emission of greenhouse gases that increase temperature and AED. In fact, both factors are not independent from each other, as land cover change may affect land ET and AED [15], while an increased AED may also affect ET and therefore the availability of water resources for other uses (e.g. reservoir storage) [36]. Here, we discuss the second factor that is linked to the rise in temperature and AED, since the first factor is very complicated and has strong regional differences.

AED has strong agronomic and hydrological implications [37]. AED is the maximum quantity of water that would evaporate under unlimited water availability and no-resistance factors from soil and vegetation or when resistance factors are not temporally and spatially varying [39,40]. AED depends on the radiative and aerodynamic components, and fully physically based AED models (i.e. the Penman equation) based on four main meteorological drivers, air temperature, radiation, atmospheric humidity and wind speed, are necessary to fully capture the AED spatial and temporal changes [41–44].

There is no scientific consensus on the role of AED in agricultural, ecological and hydrological drought severity. Accordingly, it is important to assess this issue prior to the use of drought metrics that include AED to evaluate possible changes and future projections. Nonetheless, under climate change, AED can be a powerful driver of drought severity [39,40], with multiple effects (electronic supplementary material, figure S5). AED enhances evaporation from soils and water bodies and transpiration from plants. This is particularly relevant during periods of precipitation deficits in which increased AED contributes to reducing the water resources available to plants and humans via enhanced ET [17]. Moreover, AED has direct and indirect impacts on plants under conditions of soil moisture deficit; higher AED increases plant hydraulic stress and the risk of xylem embolism and plant mortality [45]. No matter how much water is in the soil, increased AED can cause plants to take in less carbon and do less photosynthesis [42,43].

AED's diverse effects are complicated by the fact that the influences of AED on droughts vary, depending on drought type (hydrological versus agricultural and ecological), climate characteristics (i.e. water-limited versus radiation-limited regions) and precipitation variability [41]. Thus, during periods of available soil moisture, the effect of AED on plants is usually positive, as high AED correlates with high temperature and radiation, favouring photosynthesis and carbon uptake. On the other hand, when soil moisture is low, higher AED makes plant stress worse. Moreover, although ET is slight in drought periods, any water loss caused by higher AED can be important to guaranteeing plant water availability. Also, evaporation from soils and water bodies can exacerbate hydrological droughts during periods of precipitation deficits, which is a particular problem in reservoirs of dry and semi-arid regions [36]. Therefore, there is scientific consensus that an increase in AED may have an effect on different types of drought, but it is complicated and hard to figure out.

Recent studies suggest using AED as a single variable to assess changes in agricultural and ecological drought severity [46], given the complementary relationship between AED and ET [47]. It is well established that under strong land-atmosphere coupling, high AED could be indicative of low soil moisture. Nonetheless, this assumption is hindered by the notion that other factors may also cause an increase in AED. These factors may include atmospheric circulation (e.g. warm advections) and radiative forcing, which are independent of soil moisture content. Therefore, to generate synthetic metrics that are considered proxies of agricultural and ecological droughts and even hydrological droughts, it is reasonable to assume that AED is combined with variables that also provide information on water supply, water availability or water use. Still, there is no agreement on how to determine the best metric that captures the role of AED in drought severity.

Different approaches have been proposed to assess the role of AED in drought severity (electronic supplementary material, figure S6), including the difference between precipitation (P) and AED [48] (i.e. the standardized precipitation evapotranspiration index [SPEI]) and the difference between actual ET and AED [49] (i.e. the standardized evapotranspiration deficit index [SEDI]). The SPEI represents a climate balance between water supply and water demand, which provides information on the water stress of a system with particular implications on vegetation [16]. However, it also shows high correlations with hydrological droughts [50,51]. SEDI represents the transpiration deficit, which is the difference between the water that plants transpire and the water that the atmosphere demands. This difference is the quantity of water that is not available to supply to the atmosphere and determines plant stress. Transpiration deficit is a primary driver of the spatial distribution of the main world biomes [52], and from agronomic and ecophysiological perspectives, it strongly controls plant stomatal conductance, photosynthesis and carbon uptake by plants, given that it focuses on the analysis of agricultural and ecological droughts.

In recent years, the difference between P and ET (P-ET) has also been widely used to assess changes in aridity and drought severity [53,54]. Nevertheless, P-ET shows some limitations in assessing drought severity since, while the difference between precipitation and ET is widely used to determine anomalies in the water budget at basin scale, which is useful to assess hydrological droughts, this metric has important limitations in assessing the impacts on plants (i.e. agricultural and ecological droughts). This is supported by two reasons: (i) P-ET would not be a good marker for increased plant water stress associated with a higher AED increase in water-stressed regions because ET tends to P in these areas, so an increase in AED would not cause changes in ET, and P-ET would not vary as it would usually be close to zero and (ii) several world biomes respond to droughts at short timescales [55], but P-ET is only useful to assess the water budget over the long term, since the availability of water may not depend on the short-term precipitation but on the precipitation recorded over the previous humid season (electronic supplementary material, figure S7). Therefore, P-ET should be used to measure drought severity over long time periods (annual or longer) rather than short ones, but these long periods are not useful to assess ecological and agricultural drought in ecosystems that respond to short drought timescales, which are the majority on a global scale [55].

### (c) Recent drought trends based on metrics that include atmospheric evaporative demand and evapotranspiration as proxies of agricultural and ecological droughts

**Figure 3.**
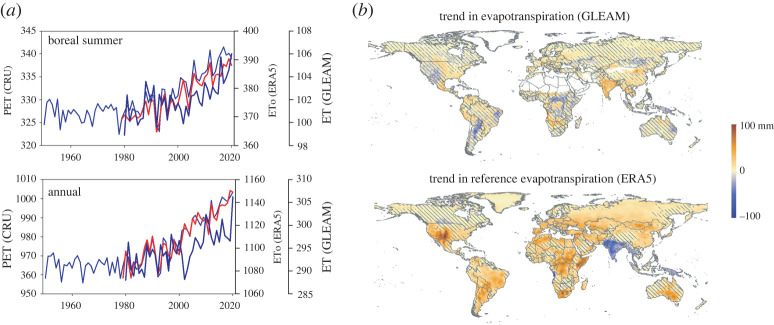
(*a*) Evolution of the boreal summer and annual AED as measured by PET (based on the CRU dataset, blue line), reference evapotranspiration (ETo, based on the ERA5 dataset, red line) and land evapotranspiration (ET) from the GLEAM dataset. (*b*) Spatial distribution of the magnitude of change in annual ET based on GLEAM dataset and AED based on ERA5 dataset (change in mm from 1980 to 2020). Overlay indicates areas with statistically non-significant trends. (Online version in colour.)

Temperatures have risen sharply in recent decades, while relative humidity has fallen as a result of differential warming between land and oceanic regions, as well as land-atmosphere feedbacks [56]. As a consequence of these changes, a general increase in AED has occurred, which is consistent across different datasets and has affected most of the world since the 1980s (figure 3). The evolution of the ET based on a database generated by a model that uses different climate information and remote sensing observations (see details below) also shows a general increase, although more limited than the AED since the evolution of the ET does not depend exclusively on demand but on soil water availability.

As the effects of AED on drought severity are complicated, it is hard to determine whether observed increases in AED over the past four decades would enhance drought severity. Therefore, we present an analysis of the temporal evolution of different drought metrics that includes AED, ET or both, for the period 1980–2020, in which there is available data on AED and ET, and where a sharp increase in AED has also been identified. This leads to the hypothesis that an increase in AED would have an impact on enhancing drought severity, with particular plant implications (agricultural and ecological droughts), since AED has a more limited effect on the severity of hydrological droughts [57,58].

Initially, we tested the possible uncertainties related to different available climate datasets since previous studies have stressed the important differences in climate trends that can be found between studies as a function of the datasets used [59]. Here we have used five global precipitation databases, two AED databases and one ET database. These databases are described in the electronic supplementary material, table S1. The FAO56 Penman-Monteith equation was used to calculate the AED from ERA5 using data on temperature, solar radiation, wind speed and relative humidity [40]. ET was obtained from the GLEAM dataset [60]. Assessment of the quality of ET modelling approaches reveals important uncertainties given the multitude of complex processes involved in land ET, which are directly and indirectly connected with climate variability and trends [61]. Nevertheless, here we have chosen the GLEAM model given its current wide use by the scientific community [62–64] and the generally good agreement found between the model outputs and ET observations from eddy-covariance methods worldwide [65]. We understand that the use of a single ET database can introduce additional uncertainty in our assessment. Nevertheless, we preferred to use a single widely used and validated database than to merge other uncertain products. The modelling scheme and the diverse databases that are used to generate the GLEAM dataset can be consulted in Martens *et al*. [65]. The use of two different AED databases on drought trends shows small uncertainty, as observed with the use of the CRU and ERA5 AED in the calculation of the SPEI (electronic supplementary material, figure S8). Nevertheless, even during the 1980–2020 period, there are important uncertainties in global precipitation data, as shown by differences in SPI trends in different datasets. These differences are particularly stronger in poorly covered areas of Africa, South America and Asia. Therefore, the main data uncertainties related to the global assessment of drought trends are still related to precipitation data.

For our assessment, we selected precipitation data from the GPCC dataset, as it uses the highest number of meteorological stations. We preferred to use a dataset based on the highest possible density of observations rather than merge products based on observations, reanalysis and satellite estimations that could bias the assessment during 1980–2020. In any case, there is not a perfect solution to this global problem, and it is necessary to stress that drought trends show uncertainties related to the choice of the precipitation product, even more so given the dominant worldwide reduction of precipitation observatories in the last decades. We used ERA5 data for AED [66] since trend uncertainty for AED is much lower than that of precipitation. The different standardized indices were calculated on a timescale of three months because the period of study covers only 40 years and longer timescales would produce datasets with few drought events, which would not provide a robust analysis. In order to identify drought events, the threshold in the different indices was set at zero.

**Figure 4.**
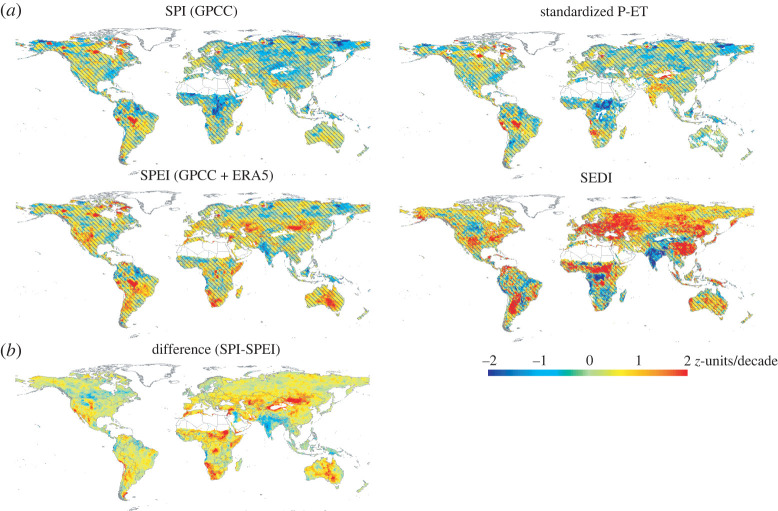
(*a*) Trends in drought event magnitude between 1980 and 2020, based on various drought metrics. Drought events were identified by means of indices on a three-month timescale, with a threshold of zero z-unit. (*b*) Differences between the trends using SPI and SPEI. Colour scale is the same for the different plots, and in all maps, the overlay indicates areas with statistically non-significant trends. (Online version in colour.)

As illustrated in figure 4*a* based on the GPCC dataset, there are no dominant trends in the magnitude of drought events that can be seen with SPI and standardized P-ET on a global scale. On the contrary, SEDI shows a reinforcement of drought magnitude in large regions such as Eastern Europe and Eastern China. SPEI shows a reinforcement of droughts in comparison with the trend of meteorological droughts in some regions. Figure 4*b* shows that the most important differences between SPI and SPEI can be found in Australia and parts of Asia and Africa.

Trends show important differences between the boreal winter and summer seasons (electronic supplementary material, figure S9), with larger areas affected by changes in indices using AED during the boreal summer. SPEI shows significant negative trends in Western North America, Australia, Southern Europe, Eastern, Central and Southern Africa and parts of South America. These trends are not identified by the SPI. During the boreal summer, SEDI decreases across much of central Europe, central Africa and much of Asia, indicating drier conditions. The standardized P-ET, on the other hand, does not reinforce dry conditions when compared with the SPI. This pattern is due to the fact that most of the regions with increased SPEI drought severity correspond to areas where soil water limitations are present and ET is controlled by precipitation. As a result, even though increased AED causes plants in water-limited regions to become more water stressed, P-ET is unable to detect this response.

The evolution of the percentage of the global area affected by severe, moderate and mild drought based on different drought metrics on a three-month timescale shows no apparent trends with the SPI and standardized P-ET (electronic supplementary material, figure S10). The SPEI shows an increase in the drying conditions in the last decade, while the SEDI shows a clear increase since 2000. The observed trends in metrics like SPEI and SEDI indicate an increased imbalance between what the atmosphere demands and the existing water availability (measured by either precipitation or ET), particularly during the warm season. These results suggest that the observed increase in AED in recent decades (figure 3) is affecting drought severity. It is difficult to estimate how much of an impact this has, although this drought increase suggested in response to enhanced AED agrees with the assessment in the recent AR6 IPCC report, which has stressed: ‘*Warming over land drives an increase in atmospheric evaporative demand and the severity of droughts (high confidence)*’ (Chapter 8, Executive Summary, [10]), particularly in western USA, the Mediterranean region, central Chile, southwestern Australia and southern Africa. Thus, the report suggested that ‘*it is very likely that anthropogenic factors have influenced global trends in aridity, mainly through competing changes in evapotranspiration and/or atmospheric evaporative demand due to anthropogenic emissions of GHG and aerosols*' (Chapter 8, §8.3.1.6, [10]). The AR6 IPCC report also suggests an increase of drought severity associated with declining soil moisture as consequence of enhanced ET given higher AED, with implications for agricultural and ecological droughts (Chapter 11, Executive Summary, [26]), particularly in some of the regions in which the drought indices assessed here show a decline (West, Central, West Southern and East Southern Africa, East and East Central Asia, Southern Australia, the Mediterranean, Western and Central Europe, Western North America and North-Eastern South America (Chapter 11, Large Tables, [26])).

Thus, this enhancement of agricultural and ecological droughts suggested by metrics that include AED in formulations is in agreement with several studies based on impact data. Increased AED has resulted in more frequent and severe episodes of tree defoliation and mortality associated with drought over the last few decades [67], as well as a crop yield decline relative to current potential [68,69]. These results suggest that although there have been no substantial changes in precipitation deficits, the severity of agricultural and ecological drought events has increased in the last four decades, which is associated with an increase in AED, particularly during the warm season in some mid-latitudinal and subtropical areas. This assessment agrees with other studies that used different databases and drought measurements, such as the Palmer drought severity index (PDSI) [6,70,71].

### (d) Recent changes in hydrological droughts

Although the available literature on climate-based drought indices shows a general high relationship with hydrological drought indices [72,73], it also has some limitations in assessing hydrological drought severity, which basically depends on the anomalies in streamflow, reservoir storage, lake levels and groundwater. Assessing the recent trends in hydrological droughts is much more difficult than the assessment of agricultural and ecological droughts due to the limited availability of data in these sources, as well as the key role of water regulation and human water use. Given these data limitations, we assess recent changes in hydrological droughts based on recent published literature from regional to global scales. Some recent global studies have suggested a decrease in streamflow [74] and an increased frequency of hydrological droughts [75] in some regions of the world since 1950, particularly in the Mediterranean region, north-eastern Brazil, West and South Africa, and in some basins of West North America and the Murray-Darling basin in Australia. As a result, anthropogenic climate change has been linked to changes in hydrological drought patterns around the world [76]. Thus, the assessment of the recent AR6 IPCC report has stressed this issue indicating that ‘*Increasing trends in hydrological droughts have been observed in a few AR6 regions*' (Chapter 11, Executive Summary, [26]), including the Mediterranean, West Africa, East Asia and Southern Australia.

The results of these studies seem to contradict the general assessment provided in §2, which showed no evident trends in meteorological droughts in these regions since the 1950s. It could be argued that the large increase recorded in AED would have increased ET and could explain the occurrence of more severe hydrological droughts. Nevertheless, an assessment of the influence of AED on hydrological droughts is much more difficult than for the agricultural and ecological droughts assessed in §4. The first reason is that surface and sub-surface water sources show little influence from AED, particularly in dry regions in which ET is mostly limited by precipitation amount, and the increase in AED has only slight hydrological significance [41] in comparison with precipitation changes [57,58], as runoff generation is mostly governed by precipitation and snowmelt. This does not mean that an increase in AED plays a negligible role in trends in hydrological droughts. Thus, some studies have demonstrated that AED has reinforced hydrological droughts in some regions, such as the Mediterranean [77], Southwest North America [78–80] and Australia [81], and there are even studies that identify an effect on groundwater [82]. Nevertheless, the role of AED is definitely much smaller than the influence of non-climatic factors on surface and sub-surface hydrology, including land cover change, human water abstraction and hydrological regulation, in explaining recent trends in hydrological droughts. This is clearly observed in areas of the world showing the greatest increases in hydrological drought severity in global studies [74,75]: northeast Brazil and the Mediterranean region. In north-eastern Brazil, increased hydrological drought severity is mostly related to the rapid increase in the surface area of irrigated land, which grew by more than 200% between 1996 and 2017 [83]. Also, in the Mediterranean region, most of the increase in the frequency and severity of hydrological droughts can be traced to changes in the land in the headwaters and a higher demand for irrigation [58,84–86].

Therefore, an increase in hydrological droughts has been observed in some world basins in the last few decades. Nevertheless, the effect of climate change processes is difficult to assess given the substantial regulation and use of human water in some regions. Thus, we think that climate change processes have had a lower role than other processes (land use changes and water demands) in explaining the spatial patterns and magnitude of change of global hydrological droughts. This is supported by the small influence that would be expected by precipitation given the few changes observed in SPI (§2). Moreover, the global increase in AED would not explain the spatial patterns of changes in hydrological droughts worldwide. Thus, the increased AED has been so homogeneous globally in the last few decades (figure 3), that if we were to support a dominant role of AED, an increase in the frequency and severity of hydrological droughts should be observed in more regions, particularly in those that do not show positive precipitation trends and in general, in all semi-arid regions in which the average river flows are low in magnitude and the AED very high. On the contrary, the increase in hydrological droughts has been primarily observed in regions with high water demand and land cover change (e.g. western North America, the Mediterranean, north-eastern Brazil and southern Australia), supporting the fact that the role of the AED increase in hydrological drought trends is small in comparison with other human-induced influences in these regions.

## 3. Global drought projections

### (a) Analysis of drought scenarios in response to enhanced CO_2_ emissions from CMIP6 models

The outputs of the CMIP6 experiments have recently been released, and different studies have already analysed the huge amount of data they generated to characterize drought changes in future climate scenarios. Regardless of the drought metric used, there is agreement that high emission scenarios of greenhouse gases will further increase the frequency and severity of meteorological, agricultural and ecological and hydrological droughts than low emission scenarios [87–91]. Precipitation decline in high emission scenarios is mostly recorded in southern North America and Central America, northern South America and the Amazon basin, southwestern America, the Mediterranean region, western and southern Africa and South Australia [10,91]. This spatial pattern agrees with the regions in which an increase in meteorological drought duration based on SPI and the length of consecutive dry days is projected [88,92,93], and the regions in which the main drying projections have been stressed in the last AR6 IPCC report (Chapter 11, Figure 11.18, [26]). The main increase in agricultural, ecological and hydrological drought conditions simulated by the models with variables as soil moisture and runoff tends to agree with the regions that show a higher increase in meteorological drought severity, but there are some spatial differences, because surface soil moisture and drought indices like the SPEI and the PDSI show large regions affected by drought conditions in future scenarios of high anthropogenic emissions [87,90,91,94] in comparison with drought metrics based on precipitation, P-ET, runoff and total column soil moisture. These regions include most of North America, Europe, East Asia and Australia, which would be also affected by the increases in drought frequency and severity based on drought indices including AED in formulations.

We present an assessment of drought scenarios from CMIP6 outputs that include monthly simulations of 13 CMIP6 models from the 1pctCO_2_ experiment, which assumes an atmospheric CO_2_ increase of 1% per year from preindustrial concentrations to 4× preindustrial CO_2_. The list of models used is in the electronic supplementary material, table S1. Data were resampled from the original spatial resolution of each model to 2.5° by bilinear interpolation. We focused on the same drought metrics that we used for the recent historical period: precipitation, P-AED, P-ET and ET-AED. AED was calculated by means of the FAO-56 Penman-Monteith equation using projections of solar radiation at the surface, maximum and minimum temperatures, relative humidity and wind speed. In addition, we included simulations of total column soil moisture and total runoff. Note that to obtain a global average assessment of the drought trends, we did not use the standardized version (e.g. SPI, SPEI or SEDI) and instead focused on the evolution of the surface area affected by dry conditions, quantified by a 5th percentile threshold established during the reference period from preindustrial to current (year 2021) atmospheric CO_2_. The 5th percentile threshold was chosen as it is comparable with that which we used to define severe droughts (1-in-20-year event). Thresholds were obtained independently for each of the 12 months, grid cell and model. To assess spatial changes in drought severity, we calculated standardized indices on a three-month timescale. Total column soil moisture and runoff were standardized following a log-logistic distribution, which provided good results. The series were forced to have an average equal to zero and a s.d. equal to one in the reference period [95]. With the standardized series, drought events were identified by a threshold of z-units equal to zero, and the duration and magnitude of each event were quantified. The same method used for the historical period was used to look at trends in the length and severity of droughts.

**Figure 5.**
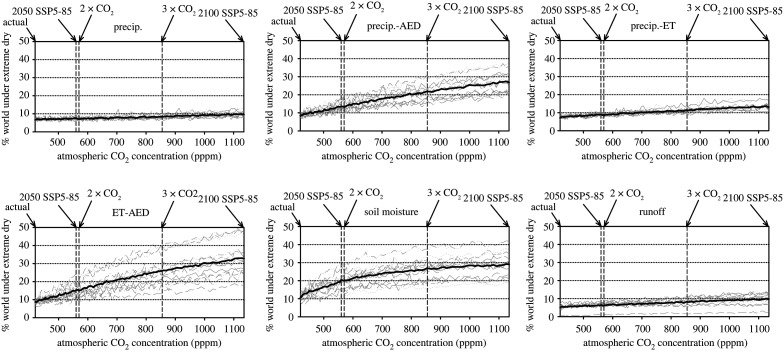
Evolution of the percentage of world areas affected by dry conditions (5th percentile) on an annual scale in response to enhanced atmospheric CO_2_ concentrations.

The evolution of the global land area affected by severe dry conditions (less than 5th percentile) in response to higher a CO_2_ shows differences between drought metrics (figure 5). Precipitation and P-ET show a small progressive increase in the global land area affected by severe dry conditions in response to enhanced atmospheric CO_2_, and there is little spread between models. The evolution of severe dry conditions for runoff closely resembles that of precipitation. The strongest increase in dry conditions in response to enhanced atmospheric CO_2_ is recorded with P-AED, total column soil moisture and ET-AED. These metrics show up to 30% of the global area affected by severe dry conditions according to atmospheric CO_2_ of the SSP5-85 scenario at the end of the twenty-first century. The largest increase was recorded with ET-AED. The spread is high between simulations of the different models, particularly for total column soil moisture and ET-AED. While precipitation and runoff follow a similar evolution, the increase in severe dry conditions is much higher for total column soil moisture. This suggests some evolution not strictly related to precipitation totals. There are also important differences in trends between the boreal winter and summer seasons (electronic supplementary material, figures S11 and S12). As expected, the main increase in the global areas impacted by dry conditions was recorded during the boreal summer. This is shown by three metrics: P-AED, ET-AED and total column soil moisture.

**Figure 6.**
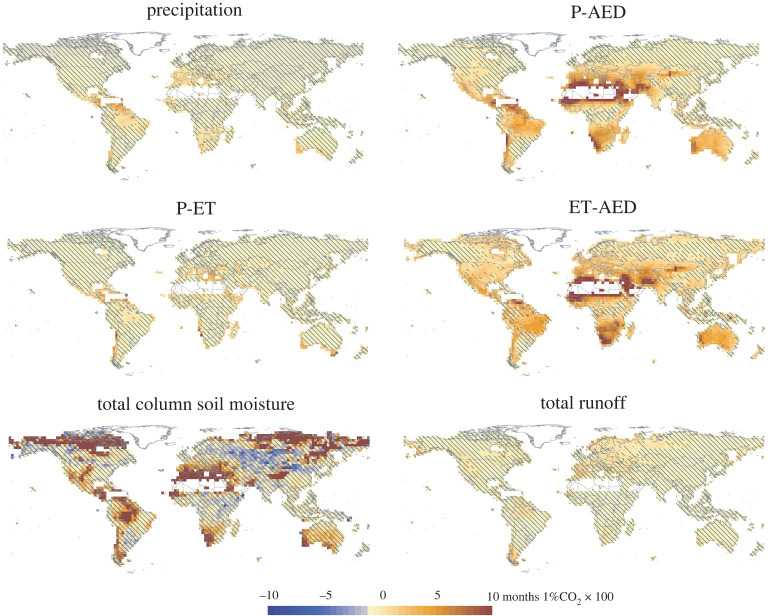
Spatial patterns of the change in drought duration between a scenario of preindustrial CO_2_ concentrations and one of atmospheric CO_2_ concentrations corresponding to the SSP5-85 scenario for the year 2100. The magnitude of change represents the median of the 12 models. Stripes correspond to areas in which less than 75% of the models show statistically significant changes. (Online version in colour.)

Spatial trends in the evolution of the duration of drought events show clear spatial differences between drought metrics (figure 6). A significant increase in the duration of meteorological droughts in response to enhanced atmospheric CO_2_ concentration has been recorded in the regions where models project a precipitation decrease like central, northern and southwestern South America, the Mediterranean region, South Africa and southwest Australia. Curiously, these drought trends are stronger than the trends in drought duration projected by P-ET and runoff. This suggests that ET projections in response to high atmospheric CO_2_ would reduce drought duration in comparison with precipitation projections. The CO_2_ fertilization influence in the CMIP models could be a determinant in this process (see discussion below). The spatial patterns of trends with P-AED and ET-AED are similar, although the areas that show significant trends in ET-AED evolution are larger (also, note the possible overestimation of ET deficit given the fertilizing effects of CO_2_). With the exception of the Amazon region, areas that show a higher increase in drought severity are characterized by semi-arid to sub-humid conditions. Also, areas affected by the increase in drought severity based on P-AED are similar to total column soil moisture, except in central Australia and some regions of central Asia and the Sahel. On the contrary, the total column soil moisture shows an increase in drought duration in high latitudes of the Northern Hemisphere and in the Himalaya. This pattern may be related to permafrost melting. Similar spatial patterns are identified with the trends in the magnitude of drought events in response to enhanced atmospheric CO_2_ (electronic supplementary material, figure S13), although this magnitude is much higher for P-AED than for other metrics, and for water-limited regions than other regions. In general, all these results are in agreement with recent studies based on high emission scenarios (SSP 5-85) from CMIP6 models that have compared drought projections using a variety of drought metrics [90,94], and with the assessment of ecological and agricultural drought projections in the last IPCC report. Although in the AR6 IPCC report, the assessment of ecological and agricultural drought projections was primarily based on the total column soil moisture, which is suggested to decline less than drought indices that use AED in calculations [96], the report shows a large increase of the frequency and severity of drought episodes based on the total column soil moisture for high global warming scenarios (Chapter 11, fig. 11.19i, [26]), showing an agreement with what drought indices as the SEDI and the SPEI project for these high global warming scenarios. Thus, the Chapter 8 of the AR6 IPCC report stressed: ‘*there is high confidence that soil moisture will decline in semi-arid, winter-rainfall dominated areas including the Mediterranean, southern Africa, south-western North America, southwestern South America, and south-western Australia, as well as in Central America and the Amazonian basin…These same regions are likely to experience increases in drought duration and/or severity (high confidence). The magnitude of expected change scales with emissions scenarios (high confidence)*’ (Chapter 8, §8.4.1.6, [10]).

### (b) Critical assessment of future drought projections

If quantification of drought changes over the last few decades is still difficult, the assessment of the drought projections under future climate change scenarios is even harder given the limitation of earth system models (ESMs) to reproduce certain key physical and biological processes that are still poorly understood. In general, there is strong agreement on a future high increase in AED in most CMIP6 models in most regions of the world [41,95], associated with the projected sharp increase in temperature and decrease in relative humidity in continental areas [97]. These projections would support enhanced agricultural and ecological drought severity, at least in regions affected by precipitation decreases and also in regions in which no changes in precipitation are projected. Nevertheless, there are still important uncertainties with the precipitation projections. As a result, when comparing long-term precipitation trends with observations, CMIP6 models are considerably limited [98]. Given the main importance of precipitation on the severity of different drought types, this needs to be taken into account when assessing the goodness of future drought projections. Furthermore, the CMIP6 models exhibit significant bias in low precipitation values [99], which are the most relevant for assessing meteorological droughts.

In addition to the uncertainty of precipitation projections, there are other important components that may play a role and are still under scientific debate. Some studies suggest that drought severity could be reduced by the response of leaf stomata conductance to increased atmospheric CO_2_ concentration (a CO_2_) (CO_2_ fertilization) [100,101], since this could limit plant transpiration. Nevertheless, assessment of future drought projections on metrics that include ET in their calculations should also be carefully interpreted, as they could be biased in different ways: (i) due to overestimation of the CO_2_ fertilizing mechanism by ESMs, ET limitation would increase the difference between ET and AED, suggesting increased drought severity based on this metric, even in areas with higher precipitation and soil moisture; (ii) in P-ET, it would underestimate plant water stress given the same mechanism. As a result, it is not logical to assume that plant stress conditions will not worsen in water-limited regions where models predict precipitation to decrease or remain unchanged and where ET is not expected to decrease due to the significant rise in AED predicted by the simulations. In these regions, although ET will not change given soil moisture limitations, AED will undoubtedly increase plant water stress.

It is necessary to stress that the effect of a CO_2_ on leaf stomatal conductance is still affected by strong uncertainties and influenced by other factors, including the radiative effects of CO_2_ [61]. Moreover, ESMs show substantial overestimation of this effect [102] that could bias drought estimations based on metrics that include ET or soil moisture, but also metrics based on AED and precipitation could be affected by land-atmosphere feedbacks. Thus, ET limitation by increased a CO_2_ contradicts observations since ET has increased in the last few decades [103], a period characterized by a high increase in a CO_2_; there are also studies supporting the fact that ET has been primarily driven by climate trends, with only a minor role played by a CO_2_ [104]. Moreover, climate warming may have an impact on plant processes that are not included in ESMs, such as water loss from leaf cuticule [105,106], which is independent of stomatal responses to a CO_2_. Finally, land use and vegetation changes severely affect land transpiration, and these are either highly uncertain in current ESMs (e.g. the leaf area index [107]) or not included [108]. All of these processes mean that evaluating future drought projections based on metrics using ESM simulations of ET should be approached with caution [61], and this caution should also affect soil moisture and runoff.

Some authors have suggested that including AED in drought severity metrics could overestimate agricultural and ecological and hydrological drought projections, particularly in water-limited regions [96], based on the fact that AED is higher than ET in these regions. Nevertheless, as emphasized above, the role of AED in agricultural and ecological drought severity is not only related to its influence on ET. Thus, in the assessment of agricultural and ecological droughts, AED is not intended to be used as a substitute for ET to assess drought severity. ET could be a substitute for precipitation in drought indices, as ET is the best metric providing information on the real plant water use [109] and a much better metric than precipitation or soil moisture, but AED must not be considered a substitute for ET since it is a metric of demand, not of water use. It is important to select the drought metrics to assess future drought severity, and the use of P-ET or soil moisture could underestimate agricultural and ecological droughts caused by enhanced AED, particularly in water-limited regions. In these regions ET is low, and higher AED does not mean higher ET, but stronger stress for plants.

It is important to note that the choice of statistical procedures used can have a significant impact on the results obtained when assessing future drought predictions. For example, some studies have inferred changes in drought in future climate projections by evaluating trends in the mean climate based on standardized and non-standardized metrics, and this choice may bias the assessment of drought projections. The first reason is that changes in the average values may hinder changes in the frequency and magnitude of the values located in the lower tail of the distribution [95], which are representative of water deficits (electronic supplementary material, figure S14). Thus, when assessing drought changes, it is necessary to focus on how anomalously dry conditions change, and these must be referenced to a particular period. The second reason is that comparable timescales must be used when assessing different drought metrics. For example, some studies have stressed the possible overestimation of drought severity in projections based on the PDSI in comparison with those based on soil moisture and runoff [96,101]. Although these metrics might provide different projections for simple physical reasons, they must be calculated on the same timescale to be comparable, since the PDSI is characterized by high temporal autocorrelation, which simply explains the reinforcement of drought severity in future projections in comparison with annual anomalies of other hydroclimate variables [95]. Thus, when looking at unusually dry conditions and comparable drought timescales, the different drought metrics tend to converge more in areas where future climate scenarios are predicted to be more severe [26,91,95].

It could be asked whether projections based on different physical metrics obtained from CMIP6 models are consistent with those projected by impact models that could support an enhancement of drought severity as a consequence of stronger evaporative demand. For ecological and agricultural systems, there is a wide consensus that vegetation defoliation and tree mortality will increase in response to more severe drought events associated with enhanced temperature and AED [110,111]. It is evidenced that the same is suggested for agricultural systems, in which climate warming and enhanced AED are expected to reinforce the impact of droughts [112,113]. For hydrological drought forecasts, the assessment is more complex. Based on runoff simulations, CMIP6 does not predict a significant increase in hydrological droughts, but this issue needs to be qualified. There is a clear contrast between the CMIP6 and hydrological models projections for changes in runoff and drought severity, with a larger increase in drought severity suggested by hydrological models [114–116]. This divergence is mostly explained by the inclusion of the CO_2_ fertilizing effects in the ESMs [117], which is very uncertain [61].

Finally, we would like to stress here that, regardless of the drought projections provided by various ESMs and based on different drought metrics, we think that the current debate on drought scenarios is strongly dependent on the projections made by models, which are uncertain for the reasons discussed above. Nevertheless, we could say that in the future, precipitation will still be the main driver controlling drought events of any type, and even in areas in which models project higher precipitation, although less frequent, periods of precipitation deficits are expected in response to natural climate variability. Thus, some studies suggest that precipitation variability will increase in response to climate change [118,119]. In periods of precipitation deficits, it is highly probable that the severity of both agricultural/ecological and hydrological droughts will increase simply because there is a high confidence of an increased AED in response to global warming (electronic supplementary material, figure S15). In addition to the periods of precipitation deficit, higher AED would also contribute to faster soil water depletion via enhanced ET [120], and it must be noted that under conditions of low soil moisture, CO_2_ fertilization would have little effect on leaf stomatal conductance because it is controlled by soil water availability [121,122]. Thus, regardless of the uncertainties in the CO_2_ fertilizing effect on water availability, there is a high certainty that this effect would not alleviate plant water stress during periods of severe water deficit (caused by low precipitation, increased ET or both) [121,123].

Moreover, during low soil moisture periods, the projected increase in AED will certainly increase plant water stress. This can be particularly serious in humid areas in which vegetation is not well adapted to water stress [124]. This pattern has already been observed in regions that have shown higher precipitation over the last few decades, but where increased AED has caused unprecedented ecological drought events affecting the natural system. For example, North Europe was affected by a severe meteorological drought in 2018 that was strongly accentuated by high AED in the summer months and had exceptional impacts on forests [125,126]. Of course, in water-limited regions, there is no doubt that in the future, periods of precipitation deficit and higher AED will increase the severity of plant water stress, simply because the difference between the available water to transpire and the water demand by the atmosphere will increase. Defoliation and plant mortality would be increased as a result of this effect on plant hydraulics, photosynthetic capacity and carbon uptake.

Assessment of the response of hydrological droughts under scenarios of higher AED is much more uncertain. Nevertheless, we should mention that strong hydrological droughts are being recorded in response to natural precipitation variability, even in regions that have shown increased precipitation over recent decades, such as the UK [127]. As the role of AED on streamflow is much lower than that of precipitation, it is expected that the response of hydrological droughts to higher AED will not be as severe as expected in agricultural/ecological droughts. Indeed, there are uncertainties relating to the role of CO_2_ fertilization, as well as those linked to how runoff generation would be affected by vegetation changes. Nevertheless, and irrespective of the uncertain magnitude of future plant transpiration, there is little uncertainty that direct evaporation from water bodies and irrigated areas will increase in response to higher AED. During times of water scarcity, the stress on water management systems and users will be amplified by the faster depletion of available water resources. As a general rule, this phenomenon could affect any region of the world [128], but it will be more severe in water-limited areas where water supply is maintained by reservoirs, such as California [129] and the western Mediterranean [85], where the problem has been already observed in recent decades.

## 4. Conclusion

This study contributes to the current debate on recent changes in drought severity and future climate change projections. This is a very difficult topic to address given the complex interactions among different drivers (atmospheric, physiological and hydrological) governing drought from agricultural, ecological and hydrological points of view. The main results relating to recent drought trends show:

— A global increase in the severity of meteorological drought is not supported by the analysis of precipitation deficits, as only a few regions of the world show an increase in the severity of meteorological droughts.
— An increase in the severity of droughts over the last few decades appears to be solely linked to a strong observed increase in AED over the last four decades, with significant implications for agricultural and ecological droughts.
— Increases in the frequency and severity of hydrological droughts can be traced in part to human activities such as land use change and agricultural intensification (e.g. the Mediterranean area, Northeast Brazil).

The assessment of projections of any drought type in future climate scenarios is more complex and uncertain given the only partially understood role of several mechanisms (e.g. CO_2_ fertilizing effects) and the difficulties of modelling some relevant processes such as vegetation change and soil hydrology. An increase in atmospheric CO_2_ concentration is expected to lead to a variety of drought outcomes depending on the specific metric used. Increased drought severity is mostly identified in areas in which ESMs project a decline in precipitation (southern North America and Central America, northern South America and the Amazon basin, southwestern America, the Mediterranean region, western and southern Africa and South Australia), but generally in water-limited regions everywhere in which enhanced AED increases plant water stress, more severe ecological and agricultural droughts are expected. It is more difficult to predict hydrological drought trends because of the role of CO_2_ fertilizing effects on ESM runoff outputs, the limited influence of AED and uncertain scenarios for human water demand or land cover changes. Overall, regardless of the uncertainty of the ESM outputs, precipitation deficits in the future are likely to cause increased agricultural, ecological and hydrological drought severity as a consequence of higher evaporative demand, which could have important implications.

## Data Availability

The data used in this article are available in different public sources that are cited in the manuscript. Additional information can be found in the electronic supplementary material. The data are provided in the electronic supplementary material [130].
